# Niobium-Containing Phosphate Glasses Prepared by the Liquid-Phase Method

**DOI:** 10.3390/ijms26010161

**Published:** 2024-12-27

**Authors:** Minori Takahashi, Shota Shiraki, Sungho Lee, Akiko Obata

**Affiliations:** 1 Graduate School of Engineering, Nagoya Institute of Technology, Gokiso-cho, Showa-ku, Nagoya 466-8555, Japan; m.takahashi.494@nitech.jp; 2 National Institute of Advanced Industrial Science and Technology (AIST), 205 Sakurazaka-4-chome, Moriyama-ku, Nagoya 463-8560, Japan; tk22006-5785@sti.chubu.ac.jp; 3 Department of Applied Chemistry, College of Engineering, Chubu University, 1200 Matsumoto-cho, Kasugai-shi, Aichi 487-8501, Japan

**Keywords:** phosphate glasses, niobium, liquid-phase method, bioactive glasses, ion elution

## Abstract

Phosphate invert glasses (PIGs) have been attracting attention as materials for bone repair. PIGs have a high flexibility in chemical composition because they are composed of orthophosphate and pyrophosphate and can easily incorporate various ions in their glass networks. In our previous work, incorporation of niobium (Nb) into melt-quench-derived PIGs was effective in terms of controlling their ion release, and Nb ions promoted the activity of osteoblast-like cells. In the present work, a liquid-phase method was used for synthesizing Nb-containing PIGs, as this method allows us to prepare a glass precursor solution at room temperature, which can be attributed to improved glass-shape design. Nb-containing PIGs were successfully prepared, and their ion release behavior was controlled by changing the Nb content in the PIGs. The functions of Nb varied according to its content. For example, in the case of PIGs containing a larger amount of Nb, Nb acted as both the network modifier and former while also inducing the formation of chain-like structures. These glasses possessed a gradual ion release in a tris-HCl buffer solution. Cotton-wool-like structured scaffolds were fabricated using the synthesized Nb-containing glass using a wet-spinning method. Because the scaffolds possess excellent flexibility and controllable ion release, they are good candidates for new biomaterials.

## 1. Introduction

Glass has been extensively studied as a material for bone repair. The most common type of glass is silicate glass, since 45S5-type bioactive glass, which is a silicate system containing a minor amount of phosphate, has been developed as the first “bioactive” material [1,2,3,4]. On the other hand, phosphate glass also has been attracting attention as a new type of bioactive glass [5,6,7,8]. Phosphate glass is classified according to phosphorus content as ultraphosphate glass (P_2_O_5_ > 50 mol%), metaphosphate glass (P_2_O_5_ = 50 mol%), polyphosphate glass (P_2_O_5_ < 50 mol%) and phosphate invert glass (PIG, P_2_O_5_ ≤ 40 mol%) [9,10,11]. The PIGs are composed of ortho-phosphate (Q_P_^0^) and pyro-phosphate (Q_P_^1^) and a larger amounts of network modifiers than network formers. The modified ions in the glass-network structure are Q_P_^0^- and Q_P_^1^-bonded via non-bridged oxygen, indicating a highly ion-bonded state [12]. The glasses have a high flexibility in chemical composition because it is easy to incorporate several types of elements into the phosphate system [13,14,15,16,17].

Ions dissolved from glasses and ceramics have been reported to have a variety of effects on bone repair [18,19,20]. For example, calcium (Ca) ions improve osteoblast differentiation and calcification [21], while magnesium ions increase bone strength and promote osteoblast adhesion, proliferation and differentiation [22,23]. These inorganic ions have appropriate amount ranges for exhibiting such positive effects on bone formation. Therefore, it is important to control the chemical durability of the glass to provide an appropriate amount of inorganic ions to bone-forming cells. Because PIGs consist of pyrophosphate and orthophosphate groups and possess easier chemical durability control than the other phosphate glass systems, they are expected to be a good candidate for the material providing therapeutic ions [13,14,15,16,17]. For example, our group has reported that the introduction of intermediate oxides such as titanium (Ti) or niobium (Nb) into PIGs prepared by melt-quenching improves the chemical durability of the glass [17]. We also revealed that Nb ions promoted the differentiation and mineralization of mouse osteoblast-like cells [24]. Thus, Nb plays a role both in controlling the chemical durability of PIGs and in enhancing their bone-forming ability.

We have recently succeeded the preparation of Ti-containing PIGs by a liquid-phase method [25]. This method allows us to obtain amorphous phosphate materials by mixing phosphate solution and cation solution [26]. A glass precursor solution can be prepared at room temperature through the method, which is attributed to excellent flexibility in regard to glass-shape design. Although there have been several reports on glasses with a PIG composition synthesized by this method [26,27,28], we have shown for the first time that ion elution can be controlled by introducing intermediate oxides into glasses prepared by this method. The prepared Ti-containing PIGs exhibited a controlled ion release depending on the amount of the introduced Ti; this is because TiO_2_ is one of the intermediate oxides, and some of Ti in the glasses functions as a network former crosslinking pyrophosphate and/or orthophosphate group. As above-mentioned, because Nb ions have been found to promote some activities of osteoblast-like cells [24], we expected the introduction of Nb instead of Ti to be effective for not only controlling the chemical durability but also enhancing the bone-forming ability of the PIGs prepared by the liquid-phase method.

The shape of a material is an important factor in its use as a biomaterial. The cotton-wool-like structured materials possess good flexibility and excellent handling. These materials contain a large space (pore) for cell migration and nutrition penetration, which can enhance tissue regeneration by cells in the body [29,30]. Based on these reports, we prepared cotton-wool-like structured materials consisting of the Nb-containing PIGs using a wet-spinning method (WS). In the WS method, normally polymer is dissolved in a solvent that dissolves the polymer well (good solvent) and extruded into another solvent in which the polymer is less soluble (poor solvent) to fabricate fibers. In this study, a mixture of the glass precursor synthesized with the liquid-phase method and polyvinyl alcohol (PVA) was shaped into a fibrous structure with the WS method. PVA is a biodegradable polymer known for its hydrophilic and biocompatible properties and is widely used in the biomaterials field [31,32].

In the present work, Nb-containing PIGs were synthesized by a liquid-phase method. The influences of Nb introduction on the glass-network structure and ion-releasing behavior of the glass were evaluated. In addition, we fabricated fibrous materials with a cotton-wool-like structure using the Nb-containing PIGs. Finally, mouse osteoblast-like cells (MC3T3-E1) were cultured on the fabricated fibrous materials to assess their cell compatibility. This is the first report of the fabrication of PIG-containing fibers by the liquid-phase method combined with WS.

## 2. Results

### 2.1. Glass Structure of Prepared Samples

**Figure 1:**
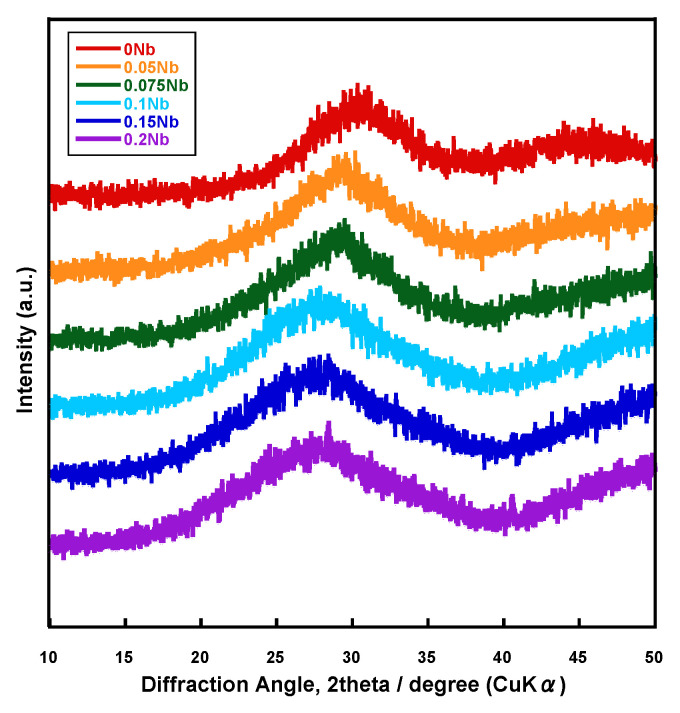
XRD patterns of the samples.

Figure 1 shows the X-ray diffractometry (XRD) patterns of the prepared samples named *x*Nbs (*x* is the concentration of niobium chloride in the solution used for the synthesis, 0~0.2). Halo patterns originating from amorphous phases were observed for all of the samples.

Table 1 shows the composition of *x*Nbs as determined by energy dispersive X-ray spectroscopy (EDS), and Figure S1 shows the EDS spectra of *x*Nbs. The contents of P_2_O_5_ and K_2_O were almost the same among the samples and were 33~38 mol% and 4~10 mol%, respectively. On the other hand, the contents of CaO and Nb_2_O_5_ varied widely; the content of CaO decreased with increasing Nb_2_O_5_. In particular, the composition changed significantly between 0.075Nb and 0.1 Nb; the contents of Nb_2_O_5_ drastically increased and almost became even with that of CaO for 0.1 Nb.

**Figure 2:**
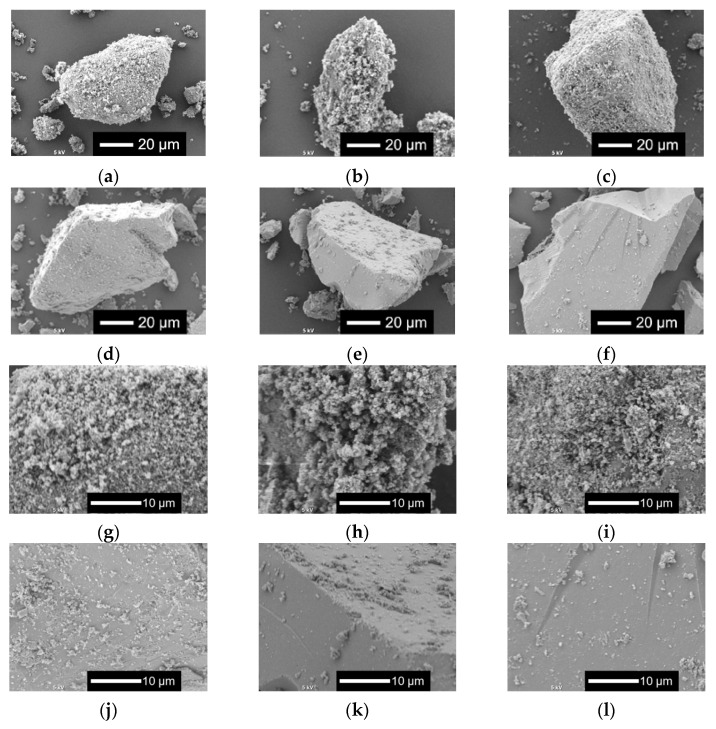
SEM images of the samples. (**a**–**f**) Low-magnified images; (**g**–**l**) high-magnified images: (**a**,**g**) 0 Nb; (**b**,**h**) 0.05 Nb; (**c**,**i**) 0.075 Nb; (**d**,**j**) 0.1 Nb; (**e**,**k**) 0.15 Nb; (**f**,**l**) 0.2 Nb.

Figure 2 shows the scanning electron microscopy (SEM) images of *x*Nbs. The surface roughness of the sample particles changed with the content of Nb in the samples. As the Nb content increased, the surface became smoother. For the samples with no or a smaller amount of Nb, tiny particles aggregated to the surfaces of the glass sample particles.

Figure S2 shows the thermogravimetry–differential thermal analysis (TG-DTA) curves of the samples, and Table 2 shows the glass transition temperature (T_g_) and crystallization temperature (T_c_) of the samples read from the TG-DTA curves. Two values were detected for T_g_ and T_c_ of 0.05 to 0.2 Nb.

**Figure 3:**
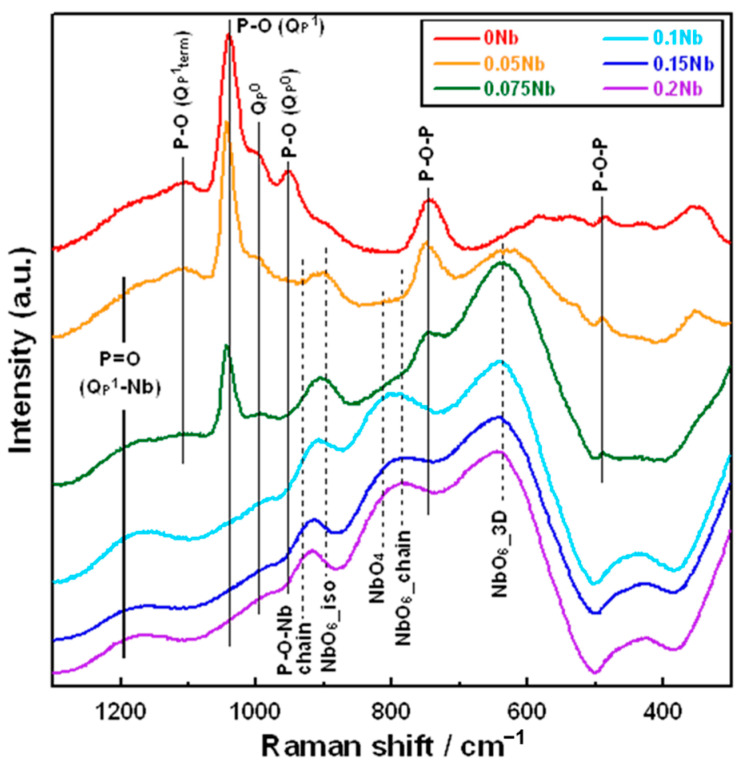
Raman spectra of the samples.

Figure 3 shows the laser Raman spectra of *x*Nb. There are obvious differences in the shape of the spectrum between the *x*Nb (*x* ≤ 0.075) and the *x*Nb (0.1 ≤ *x*) group. For 0 Nb, we observed strong peaks at 740 and 1040 cm^−1^, which originated from the Q_p_^1^ structure, and weak peaks at 950 and 990 cm^−1^, which originated from the Q_p_^0^ structure [33]. For 0.05 Nb and 0.075 Nb, in addition to these peaks mentioned above, a peak at 630 cm^−1^ derived from the NbO_6__3D structure and a peak at 900 cm^−1^ derived from the NbO_6__iso structure appeared [34].

In the *x*Nb (0.1 ≤ *x*) group, peaks related to Nb with the other structures were observed. For 0.1 Nb, peaks derived from the NbO_6__chain structure at 780 cm^−1^, the NbO_4_ structure at 810 cm^−1^ [34] and the P-O-Nb chain structure at 930 cm^−1^ were also observed [35]. For 0.15 Nb and 0.2 Nb, the peaks derived from the NbO_6__chain and NbO_4_ structures possessed a slightly stronger intensity, while the peak derived from the NbO_6__3D structure was weaker compared to that for 0.1 Nb.

**Figure 4:**
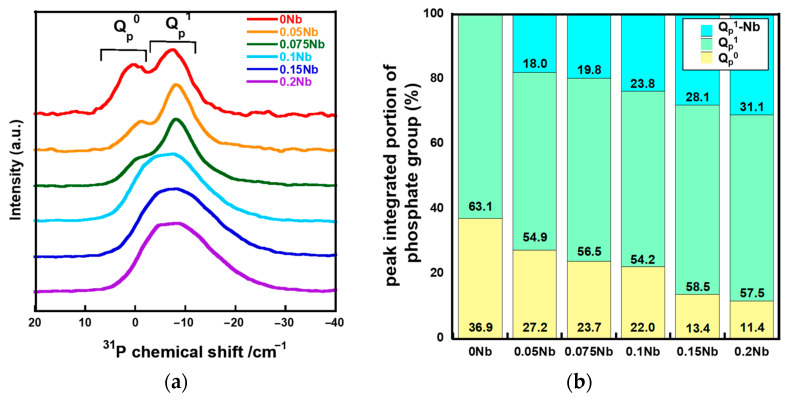
^31^P MAS-NMR results of the samples. (**a**) spectra; (**b**) fractured peak integrated portion of *Q*_P_^0^, *Q*_P_^1^, and *Q*_P_^1^-Nb.

Figure 4a shows the solid-state ^31^P magic angle spinning nuclear magnetic resonance (^31^P MAS-NMR) spectra of *x*Nbs. The spectra of 0 Nb, 0.05 Nb and 0.075 Nb exhibit peaks corresponding to Q_p_^0^ and Q_p_^1^ at around 2 ppm and -6 ppm, respectively [36,37]. For 0.1 Nb, 0.15 Nb and 0.2 Nb, a broad peak was extended to -22 ppm which corresponds to the Q_p_^2^ region. The peak in the spectrum for 0.15 Nb and 0.2 Nb exhibits an expansion that is slightly larger than 0.1 Nb. In our previous work, such expanding of the peak was observed in the ^31^P MAS-NMR spectra of the Ti-containing PIGs prepared by the liquid-phase method due to Ti-bonding to Q_P_^1^ [25]. Therefore, the ^31^P MAS-NMR spectra of *x*Nbs were deconvoluted into peaks of Q_P_^0^, Q_P_^1^, and Q_P_^1^-Nb (Figure S3). Figure 4b shows the integrated area ratios of Q_P_^0^, Q_P_^1^, and Q_P_^1^-Nb obtained from the deconvolution. The *Q_P_*^0^ peak areas decreased with the increase in the Nb content, whereas those of Q_P_^1^-Nb increased.

### 2.2. Ion Release from Samples in Tris-HCl Buffer Solution

**Figure 5:**
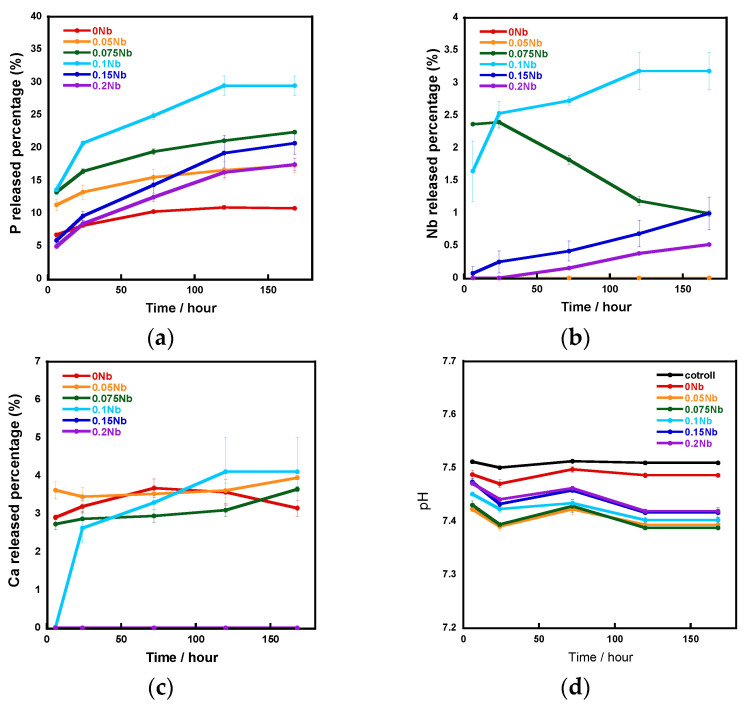
The ratio of released ions to total amount in samples during immersion in a tris-HCl buffer solution for 7 days: (**a**) P; (**b**) Nb; (**c**) Ca; (**d**) pH of the tris-HCl buffer solution.

Figure 5 shows the ion release amounts in a tris-HCl buffer solution measured using an inductively coupled plasma atomic emission spectrometer (ICP-AES) and the pH of the aforementioned tris-HCl buffer solution. The vertical axis shows the percentage of released P, Nb and Ca element amounts relative to the total amount of each element contained in the samples, which were calculated using the results of the EDS composition analysis. The release percentage of P and Nb increased with the increase in the Nb content from 0 to 0.1 and decreased with increase in that from 0.1 to 0.2. In the case of Ca release, although the percentages for 0 Nb to 0.1 Nb were similar, no release was observed for 0.15 Nb and 0.2 Nb. During the 7-day soaking, the pH of the solution was 7.4~7.5.

**Figure 6:**
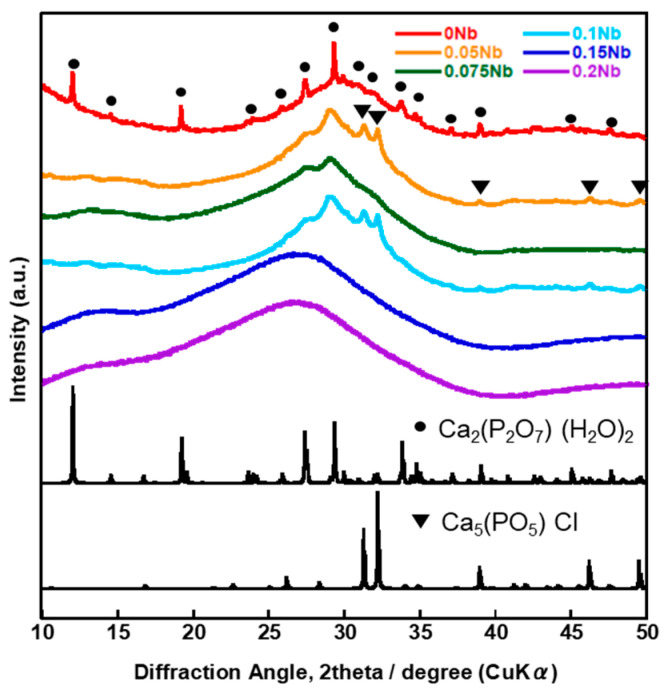
XRD patterns of the samples after immersion in tris-HCl buffer solution for 7 days.

**Figure 7:**
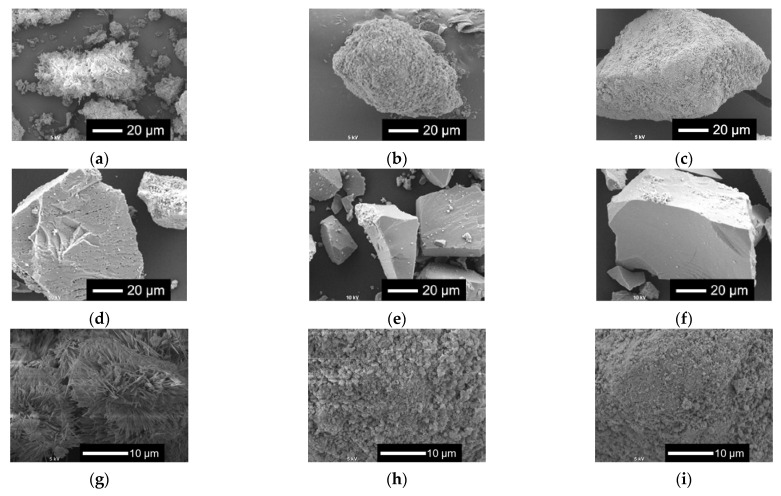

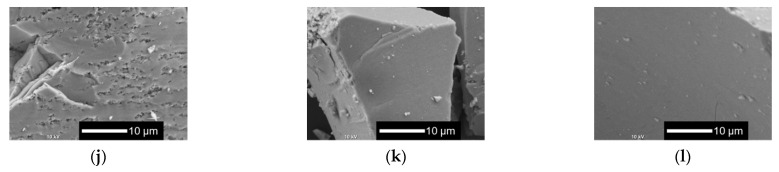
SEM images of the samples after immersion in tris-HCl buffer solution for 7 days. (**a**–**f**) Low-magnified images; (**g**–**l**) high-magnified images: (**a**,**g**) 0 Nb; (**b**,**h**) 0.05 Nb; (**c**,**i**) 0.075 Nb; (**d**,**j**) 0.1 Nb; (**e**,**k**) 0.15 Nb; (**f**,**l**) 0.2 Nb.

Figure 6 shows the XRD patterns and Figure 7 shows the SEM images of *x*Nbs after immersion in tris-HCl buffer solution for 7 days. For the XRD patterns, peaks derived from calcium-phosphate-based crystalline phases were observed for 0 to 0.1 Nb. However, in the case of 0.05 to 0.1 Nb, because their patterns contain only a few sharp peaks and predominantly consist of a broad peak, the amount of the precipitates was expected to be small. No sharp peaks were found for 0.15 and 0.2 Nb. These results were supported by the SEM observation. Although needle-shaped precipitates were observed on 0 Nb, no precipitates were observed for the other samples.

### 2.3. Structure and Ion Release Behavior of Fibrous Scaffolds

**Figure 8:**
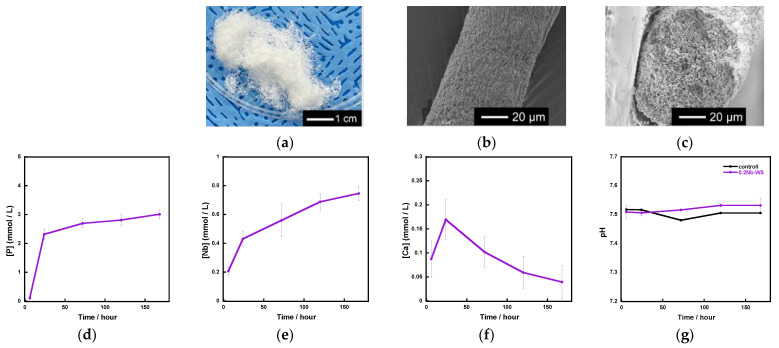
Fibrous scaffolds consisting of a 0.2 Nb-PVA composite: (**a**) appearance; (**b**) SEM image of a single fiber; (**c**) SEM image of a cross section of the fiber; (**d**–**f**) concentration of the ions released from the scaffold samples in the tris-HCl buffer solution; (**d**) P; (**e**)Nb; (**f**) Ca; (**g**) pH of tris-HCl buffer solution.

Based on the above results, we used 0.2 Nb to fabricate fibrous scaffolds because 0.2 Nb contained the largest amount of Nb among the synthesized samples and possessed a gradual ion release. Figure 8a shows the appearance of the fibrous scaffold consisting of 0.2 Nb and a polyvinyl alcohol (PVA) composite prepared by a wet-spinning method. They possessed a cotton-wool-like structure and excellent flexibility. The fiber diameter was found to be 40~120 μm from the SEM observation for 20 parts, which were randomly picked, of the samples. There were no micrometer-sized pores on the surface or inside of a single fiber (Figure 8b,c). From the results of the immersion test using tris-HCl buffer solution (Figure 8d–f), the fibers exhibited a gradual ion release of P and Nb. However, in the case of Ca, its concentration increased until day 1 and then gradually decreased. During the 7 days of immersion, the pH of the solution was about 7.5 (Figure 8g) and only changed by a small amount.

### 2.4. Cell Proliferation on the Fibrous Scaffolds

**Figure 9:**
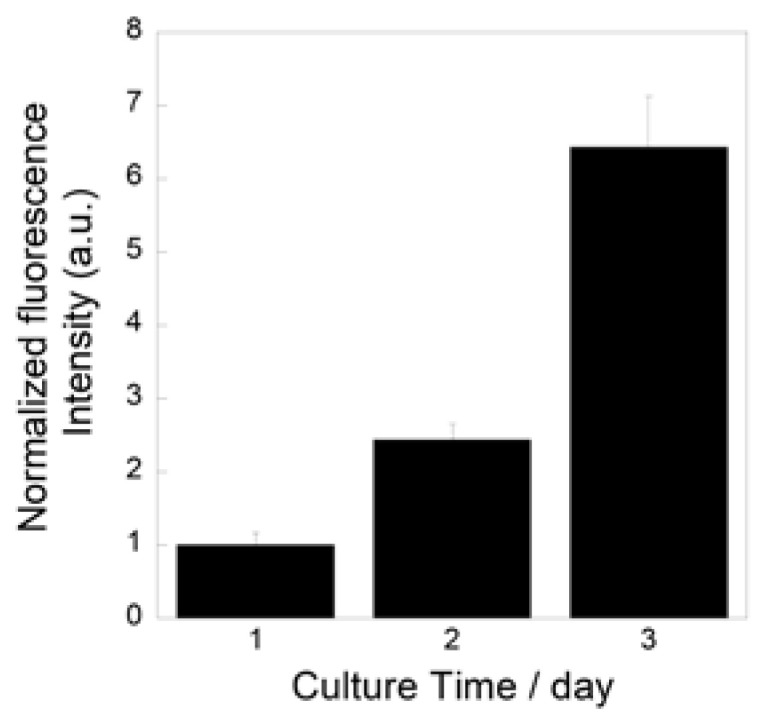
Metabolic activity levels of MC3T3-E1 cells cultured on prepared fibrous samples.

Figure 9 show the metabolic activity levels of the MC3T3-E1 cells cultured on the prepared fibrous samples. The levels increased during the culture, which indicates that the cells successfully proliferated on the samples.

## 3. Discussion

Since all of the prepared samples were found to consist of an amorphous phase from their XRD patterns, as well as to exhibit T_g_ from their DTA results, we confirmed that Nb-containing glasses were successfully prepared by the liquid-phase method. The spectra of laser Raman and ^31^P MAS-NMR (Figure 3 and Figure 4) suggest that these glasses were composed of Q_p_^0^ and Q_p_^1^, and their glass network was varied depending on the amount of Nb introduced. The Nb content in the resulting samples (Table 1) and the glass-network structures (Figure 3 and Figure 4) changed drastically when the amount of Nb introduced during synthesis (*x* value) increased from 0.075 to 0.1.

For the *x*Nb group (*x* ≤ 0.075), Raman spectra revealed that Nb formed a six coordination unit that functions as a network modifier in glass networks. The ^31^P MAS NMR spectra demonstrated that Q_p_^0^ peak shifts to the higher field side after introducing Nb. It has been reported that the chemical shift in the ^31^P MAS NMR spectrum is sensitive to the electronegativity, the charge and the radius of next-nearest-neighbor cations [38]. Nb has higher field strength than calcium. Thus, the observed peak shift must indicate the increase in the electron density surrounding the phosphorus in the Q_p_^0^ group due to the substitution of Ca, which coordinates to the Q_p_^0^ group, with Nb.

Raman spectra revealed that the *x*Nb (0.1 ≤ *x*) group contains a P-O-Nb chain structure and six and four coordination units of Nb. This indicates that Nb can act as both a network former and a network modifier. Although Nb was reported to predominantly form a six coordination unit [39], the glasses with (50-*y*)P_2_O_5_–*y*Nb_2_O_5_–50SrO (*y* = 15 and 20) and 40P_2_O_5_–20Nb_2_O_5_–40SrO systems were found to contain Nb with six and four coordination units from their Raman spectra, and the content of the four coordination unit increased with the increase in the *y* value from 15 to 20 [34]. Therefore, Nb with a four coordination unit is expected to form and act as a network former in the *x*Nb, and its content must increase with the increase in the amount of Nb incorporated. In our previous study on melt-quench-derived glasses with a CaO–P_2_O_5_–TiO_2_/Nb_2_O_5_ system, the amount of Nb with a four coordination unit in the glasses increased with increase in the amount of CaO replaced with P_2_O_5,_ and this phenomenon was also observed for Ti in the TiO_2_-sereis glasses. This suggests that the role of Nb in the glasses was similar to that of Ti [17]. We also revealed that, in the case of Ti-containing PIGs synthesized by the liquid-phase method, the amount of Ti with a four coordination unit increased with the increase in the content of Ti in the glasses [25]. Based on these reports, we expect that the Nb in the *x*Nb plays the same role as Ti in the Ti-containing PIGs and that some of the Nb forms a four coordination unit and acts as a network former. On the other hand, because Nb^5+^ exhibits a high field strength (1.26 Å^−2^) which is close to that of network-forming cations (>1.3 Å^−2^) [40], all Nb in the glass might act as a network former regardless of its coordination unit. Nevertheless, in the case of the *x*Nb (0.1 ≤ *x*) group, a large amount of Nb was incorporated into the glasses and acted as a network former.

**Figure 10:**
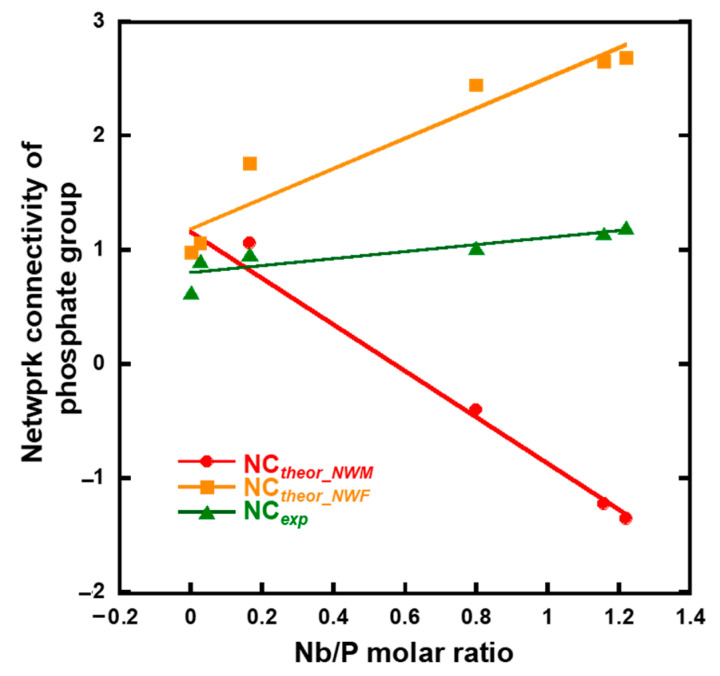
Calculated network connectivity of the samples.

The network connectivity of *x*Nb was calculated using Equations (1)–(3) (Figure 10). The experimental network connectivity (*NC_exp_*) was calculated using the deconvoluted ^31^PMAS-NMR spectra (Figure S5 and Figure 4b), assuming the presence of Q_P_*^b^* units (*b*: number of bridging oxygen atoms of P). The *NC_exp_* was calculated using Equation (1), where the number of bridging oxygen atoms (*b* value) of Q_P_^1^-Nb is two.

(1) $${NC}_{exp}=\sum_{b=0}^{3}b\;\left[Q_{p}^{b}\right]$$

The theoretical network connectivity is regarded as the number of bridging oxygen atoms per network-forming element [41]. When we assume that all Nb_2_O_5_ in *x*Nb act as either a network modifier or network former, the network connectivity of *x*Nb can be calculated in two different ways, with Nb_2_O_5_ acting as an network modifier (*NC_theor_NWM_*) or network former (*NC_theor_NWF_*), using Equations (2) and (3) [15]:

(2) $${NC}_{theor\_NWM}=\frac{3\left[P_{2}O_{5}\right]-\left[K_{2}O\right]-\left[CaO\right]-3[{Nb}_{2}O_{5}]}{\left[P_{2}O_{5}\right]}\;$$

(3) $${NC}_{theor\_NWF}=\frac{3\left[P_{2}O_{5}\right]-\left[K_{2}O\right]-\left[CaO\right]+3[{Nb}_{2}O_{5}]}{\left[P_{2}O_{5}\right]+[{Nb}_{2}O_{5}]}\;$$

where [P_2_O_5_], [CaO], [K_2_O] and [Nb_2_O_5_] are the molar fractions of phosphate, calcium oxide, potassium oxide, and niobium oxide, respectively. The *NC_exp_* value of 0 Nb and 0.05 Nb was approximately the same as *NC_theor_NWM_* and *NC_theor_NWF_*. Although the *NC_exp_* value of 0.075 Nb was still close to that of *NC_theor_NWM_*, the values of *x*Nb (0.1 ≤ *x*) gradually approached *NC_theor_NWF_*, and the differences in the values between *NC_exp_* and *NC_theor_NWM_* drastically increased with an increase in the Nb content (Figure 10). This indicates that a large number of the Nb in *x*Nb acted as a network former, especially in *x*Nb (0.1 ≤ *x*).

This is supported by the results of ^31^P MAS-NMR; a peak broadens to −22 ppm, which is considered to the region for the Q_p_^2^ structure. The deconvolution results demonstrated that the content of the Q_p_^2^-like structure (denoted by Q_P_^1^-Nb in Figure S3 increased with an increase in the Nb content. This suggests that Q_p_^1^ in the glasses is cross-linked by Nb and forms a Q_p_^2^-like structure because their Raman spectra do not exhibit peaks corresponding to the Q_p_^2^ chain structure around 700 and 1170 cm^−1^. It has been reported that ^31^P MAS-NMR peaks of P(OH)_2_(OSi) and H_4_P_2_O_7_ were observed in almost the same region, making them almost impossible to assign [42,43]. This means that the peak corresponding to Q_p_^1^ is found in the same region regardless of the P-O-Si or P-O-P bond in Q_p_^1^. On the basis of these things, the *x*Nb (0.1 ≤ *x*) group is expected to contain more long-chain structures, such as (-O-P-O-P-O-Nb-O-). This is supported by the results of the measurement of T_g_ and T_c_ by TG-DTA; T_g_ and T_c_ were observed at two different temperatures for *x*Nb. Because long-chain structures require more energy for migration and crystallization than short structures, the appearance of additional T_g_ and T_c_ is due to the formation of the above-mentioned long-chain structure. In addition, Nb_2_O_5_ might form three-dimensional cluster structures, because the NbO_6__3D structure was contained in the Nb-containing samples, as shown in the Raman spectra (Figure 3). Such structures were reported to form in metaphosphate glasses prepared by the melt-quenching method [44,45]. The cluster formation also may contribute to the appearance of two different T_g_ and T_c_. Further investigation will be needed to clarify more details about the Nb state.

The results of the immersion tests demonstrated that, in the case of *x*Nb (*x* ≤ 0.1) group, the percentage of P and Nb release increased with the increasing Nb content in the glasses (Figure 5). The formation of Ca_2_(P_2_O_7_) (H_2_O)_2_ or Ca_5_(PO_5_) Cl crystals was found for 0 to 0.1 Nb after immersion from the XRD results (Figure 6), and the needle-shaped precipitate was observed for 0 Nb from the SEM observation (Figure 7). These results indicate that 0 Nb possessed the lowest chemical durability among the prepared samples and released a large amount of ions, which induced the crystal precipitation. Because Nb exhibits a higher field strength compared to Ca, the substitution of Ca with Nb can contribute to enhancement of the chemical durability of glasses [46]. As shown in Table 1, the content of CaO in the prepared glasses decreased with the increase in that of Nb_2_O_5_. Thus, the ion release of the glasses was suppressed by Nb substitution, which contributed to the delay of the crystal precipitation with the dependence on the Nb content.

In the case of the group *x*Nb (0.1 ≤ *x*), the percentage of P and Nb release decreased with the increasing Nb content (Figure 5). Furthermore, no crystal peaks or precipitates were observed for 0.15 Nb and 0.2 Nb even after immersion in a tris-HCl buffer solution for 7 days, according to the results of XRD and SEM (Figure 6 and Figure 7). On the basis of the results of characterization on their glass structure, these samples were expected to have a long-chain structure, such as (-O-P-O-P-O-Nb-O-). It has been reported that the formation of P-O-Nb bonds increased the chemical durability of phosphate glasses prepared by the melt-quenching method [17]. Thus, with the increasing Nb content, the formation of the long-chain structure containing P-O-Nb bonds contributed to the improved chemical durability and the sustained ion-providing ability.

We successfully fabricated the cotton-wool-like scaffolds using Nb-containing glass. They possessed good flexibility and porous structures which can induce cell migration. Because the 0.2 Nb and PVA were mixed in the liquid phase before the wet-spinning process, the surfaces and insides of the obtained fibers possessed dense and homogeneous mixed structures (Figure 8b,c). They gradually released P and Nb ions in the buffer solution, and the release was sustained during the immersion (Figure 8d,e). The release of Ca ions was stopped after 1 day of immersion, and the ion concentration decreased after that (Figure 8f). On the basis of the results of the immersion test using the glass powders, the decrease in the Ca concentration is expected to be due to the precipitation of calcium phosphate-based crystals on the fiber surfaces. Another possible reason is the formation of a Nb_2_O_5_ gel on the fiber surface. The gel formation might suppress the ion release from the glass. Further investigation will be needed to clarify the state of the fiber surfaces. Because the osteoblast-like cells successfully proliferated on the fibrous scaffolds, as shown in Figure 9, the scaffolds are expected to possess a good cell compatibility. Thus, the prepared fibrous scaffolds are expected to act as good candidates for the materials used for bone regeneration due to their ability to provide therapeutic ions over a long period while having no effect on the pH of the surrounding solution.

## 4. Materials and Methods

### 4.1. Preparation of Samples

A quantity of 50 mL of an aqueous solution containing K_4_P_2_O_7_ (0.2 M, Sigma-Aldrich) (Solution A) and 50 mL of an aqueous solution containing CaCl_2_ (1 M, Wako Pure Chemical) and NbCl_5_ (0~0.2 M, Wako Pure Chemical) (Solution B) were prepared separately. Solution B was added dropwise to Solution A at a rate of 2 mL/min. The resulting suspension was filtered by suction, washed with ultrapure water and ethanol and dried at 200 °C for 1 d. The samples were indicated by *x*Nb (x: concentration of niobium chloride concentration in Solution B, 0~0.2 M).

### 4.2. Characterization of Glass Structure

The glass nature was confirmed by XRD (Philips, X’ Pert Pro α1) with a CuKα X-ray source at 3.3 °/min with a scan step of 0.26 °. The morphology of the samples was observed using SEM (JEOL, Tokyo, Japan, JSM-6000). The glass composition was determined by EDS (JEOL, Tokyo, Japan, JED-2300) (n = 3). The glass transition temperature (T_g_) and crystallization temperature (T_c_) were determined by TG-DTA (Rigaku, Tokyo, Japan, Thermoplus, TG8120) with a heating rate: 5 °C/min using 20 mg of glass powders (n = 1). The glass structure was evaluated by laser Raman spectroscopy on samples in the Raman shift region between 100 and 1800 cm^−1^ (HORIBA, Kyoto, Japan, LabRam HR800). The samples were excited by the 514 nm line of a neodymium:yttrium aluminum garnet solid-state laser with a power of 50 mW. The exposure time was 20 s and the cumulative number was 16. The ^31^P MAS-NMR (JEOL, Tokyo, Japan, JNM-ECA600II) was also used to clarify the phosphate structures in the glasses at 242.955 MHz in a 3.2 mm rotor spinning at 15 kHz. A single-pulse experiment with a 2.77 μs pulse width, 5 s recycle delay and cumulated number of 128 was performed for each sample. The chemical shift was referenced to the signal of NH_4_H_2_PO_4_ as 1.0 ppm.

### 4.3. Measurement of Ion Release from Samples in Tris-HCl Buffer Solution

Tris-HCl buffer solution was prepared by dissolving 6.118 g of tris (hydroxymethyl) aminomethane (Kishida Chemical, 99.0%) in 1 L of distilled water and adjusting the pH to 7.40 using 1 mol/L of HCl. The samples were sieved to a particle size of 32~125 μm. Seventy-five mg of the sample was immersed in 50 mL of buffer solution and maintained at 37.0 °C at 120 rpm (n = 3). At each time point (6, 24, 48, 72 h), 0.5 mL of solution was removed from each sample and 0.5 mL of fresh buffer solution was added. The collected solution was diluted 20 times, and the concentrations of P, Ca, and Nb in the resulting solution were determined using ICP-AES (Shimadzu, Kyoto, Japan, ICPS-7510). Their dissolution rates were calculated using the following equation [17]:

(4) $$Dissolution\;rate\;(\%)=\frac{1000\times (x/W_{Atom})}{\left([{Atom}_{Rate}]\times W_{sample}\right)/\left(M_{glass}\times V_{solutiom}\right)}\times 100\;\;\;$$

where *x* (ppm) is the ion amount dissolved in the tris-HCl buffer solution after soaking, measured by ICP-AES. *W*_Atom_ is the atomic weight of the ion, and [*Atom*_Rate_] is the atomic rate of the ion calculated from the nominal glass composition. *W*_sample_ (g), *M*_glass_ (g/mol), and *V*_solution_ (L) are the weight of the sample soaked, the molecular weight of the glass, and the volume of solution used for soaking, respectively. The pH of the buffer solutions were measured at each time point. The control sample was the buffer solution without any samples. The samples after soaking in the buffer solution for 7 d were washed with ethanol and dried at 60 °C for 1 day, followed by the SEM observation and the examination using XRD at 0.1 °/min with a scan step of 0.05°.

### 4.4. Fabrication of Fibrous Scaffolds and Measurement of Their Ion Release

A glass precursor solution of 0.2 Nb was prepared with the above-mentioned method. After finishing adding Solution B to Solution A, the resulting solution was centrifuged at 2100 rpm (720× *g*) for 10 min. The supernatant was collected, mixed with ultrapure water, stirred using a vortex, and centrifuged with the same condition. After repeating this process twice, the supernatant was collected and mixed with a solution of 7 wt% PVA (Mw = 85,000–124,000, Sigma–Aldrich, Tokyo, Japan) with a weight ratio of 1: 1, followed by stirring for 3 h. The PVA solution was prepared by adding PVA to water heated at 60 °C and then cooled to room temperature before being mixed with the glass precursor solution. For the wet-spinning process, the mixture solution containing PVA and the glass precursor of 0.2 Nb was placed in a syringe attached with a 30 G needle and then extruded into an acetone bath with an extrusion speed of 50 µL/min, resulting in the formation of fibers. The fibers were collected from the acetone bath and dried at room temperature. For measurement of their ion release, 20 mg of the prepared fibers were immersed in 20 mL of tris-HCl buffer solution and maintained at 37.0 °C at 120 rpm (n = 3). The ion concentration of the buffer solution was measured by ICP-AES with the above-mentioned method. The pH amounts of the buffer solutions were measured at each time point. The control sample was the buffer solution without any samples.

### 4.5. Cell Culture Tests for Fibrous Scaffolds

The prepared fibrous samples were sterilized with UV irradiation for one day. Ten mg of the samples was placed in a 96-well plate. A suspension of mouse osteoblast-like cells (MC3T3-E1 cells, Riken PRC, Ibaraki, Japan) were prepared using MEMα (Wako Pure Chemical, Osaka, Japan) containing 10 vol.% fetal bovine serum (FBS, Cosmo Bio, Tokyo, Japan) and 1 vol.% of Penicillin–Streptomycin (Gibco). The cells were seeded onto the samples at a density of 5.0 × 10^3^ cells/well (n = 4) and incubated in a CO_2_ incubator at 37 °C (humidified atmosphere of 95% air and 5% CO_2_). The culture medium was replaced with a fresh one every day.

The proliferation level of the cells after being cultured on the sample was measured using the alamarBlue cell viability reagent (Thermo Fisher Scientific, Tokyo, Japan), following instructions from the kit. Briefly, the alamarBlue reagent was diluted with the culture medium with a ratio of 1: 10 in volume; then, 110 μL of the prepared solution was added in each well and incubated in the CO_2_ incubator for 4 h. To measure the metabolic activity of the cells, the fluorescence of the medium taken from each well was measured with an excitation wavelength of 540 nm and emission wavelength of 590 nm using a multimode plate reader (PerkinElmer, Shelton, WA, USA, EnSpire).

## 5. Conclusions

Nb-containing phosphate glasses were prepared using the liquid-phase method, and their structure and ion release behavior were studied. The role of Nb in the glass network was changed depending on the amount of Nb introduced. In the case of the group *x*Nb (*x* ≤ 0.075) group, Nb acted mainly as a network modifier and was introduced into the glass network through the substitution of Ca. Although the chemical durability of the glasses was enhanced by the substitution, the glasses still released ions, which induced the precipitation of calcium phosphate-based crystals during the 7 days of immersion in tris-HCl buffer solution. In the case of the *x*Nb (0.1 ≤ *x*) group, Nb mainly acted as network former and formed long-chain structures such as (-O-P-O-P-O-Nb-O-). The chemical durability of the glasses was further improved by the formation of such chain structures. By using a combination method of the liquid-phase method and the wet-spinning method, we successfully fabricated cotton-wool-like structured materials with gradual ion-release properties. They can be good candidates for new biomaterials with the controllable ability of providing therapeutic ions.

## Figures and Tables

**Table 1:** Chemical compositions of the samples determined by EDS (mol%).

Sample Code	P_2_O_5_	K_2_O	CaO	Nb_2_O_5_
0 Nb	33.4 ± 6.3	4.4 ± 0.7	63.1 ± 5.1	N.D. ^1^
0.05 Nb	34.8 ± 2.8	4.7 ± 0.5	59.7 ± 3.0	0.89 ± 0.5
0.075 Nb	38.3 ± 1.8	4.3 ± 0.4	51.1 ± 2.0	6.3 ± 1.1
0.1 Nb	34.9 ± 0.7	4.5 ± 3.7	30.4 ± 2.4	27.9 ± 3.0
0.15 Nb	34.4 ± 0.5	9.8 ± 0.5	16.0 ± 1.3	39.8 ± 0.7
0.2 Nb	34.3 ± 1.9	8.9 ± 0.1	15.6 ± 3.5	41.8 ± 5.3

^1^ No detected.

**Table 2:** T_g_ and T_c_ of the samples read from the TG-DTA curves (°C).

Sample Code	T_g1_	T_g2_	T_c1_	T_c2_
0 Nb	544	-	594	-
0.05 Nb	530	780	905	-
0.075 Nb	561	775	-	-
0.1 Nb	511	690	774	859
0.15 Nb	510	777	859	-
0.2 Nb	520	783	862	-

## Data Availability

Data are contained within the article.

## Supplementary Materials

The following supporting information can be downloaded at: https://www.mdpi.com/article/10.3390/ijms26010161/s1.
