# Supplementary material for: Niobium-Containing Phosphate Glasses Prepared by the Liquid-Phase Method
Source: Int J Mol Sci. 2024 Dec 27;26(1):161. doi: 10.3390/ijms26010161 (PMC11720514; doi:10.3390/ijms26010161)
Supplement: Supplementary file 1 [file ijms-26-00161-s001.zip › ijms-3380306-supplementary.pdf]

Supporting information

Niobium-containing phosphate glasses prepared by the liquid-phase method

Minori Takahashi, Shota Shiraki, Sungho Lee* and Akiko Obata*

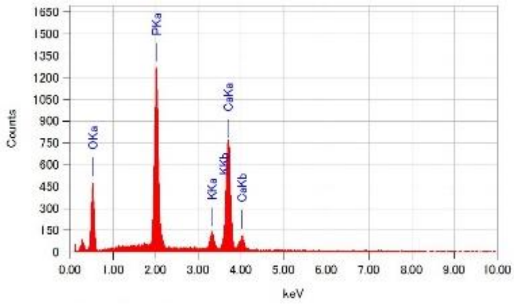

(a)

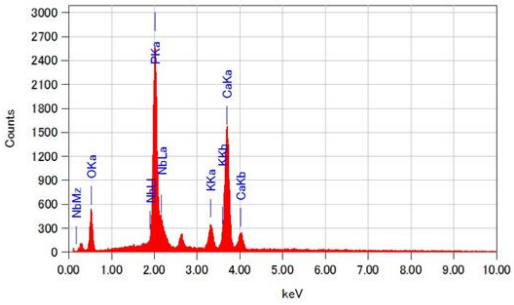

(b)

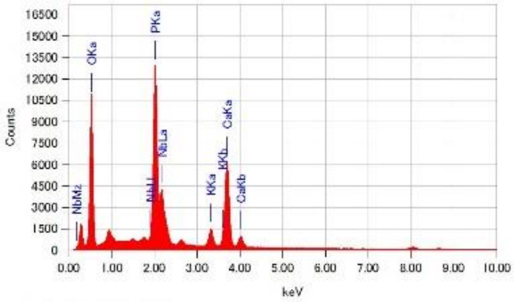

(c)

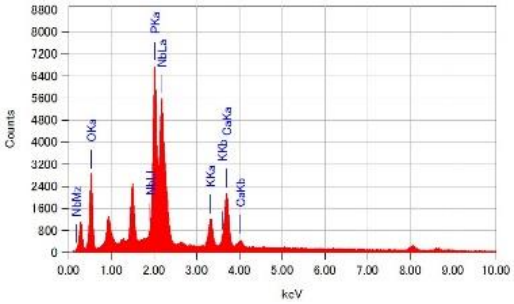

(d)

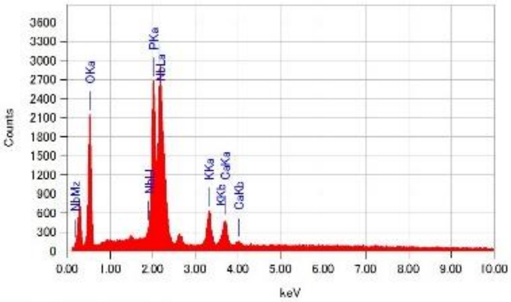

(e)

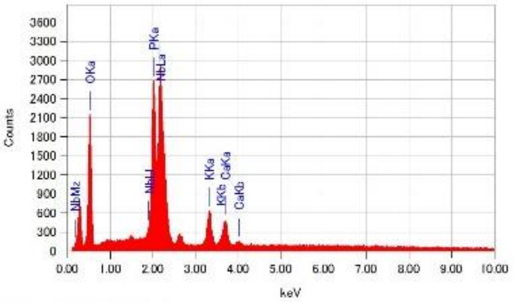

(f)

Figure S1. EDS spectra of the glasses : (**a**) 0Nb; (**b**) 0.05Nb; (**c**) 0.075Nb; (**d**) 0.1Nb; (**e**) 0.15Nb; (**f**) 0.2Nb.

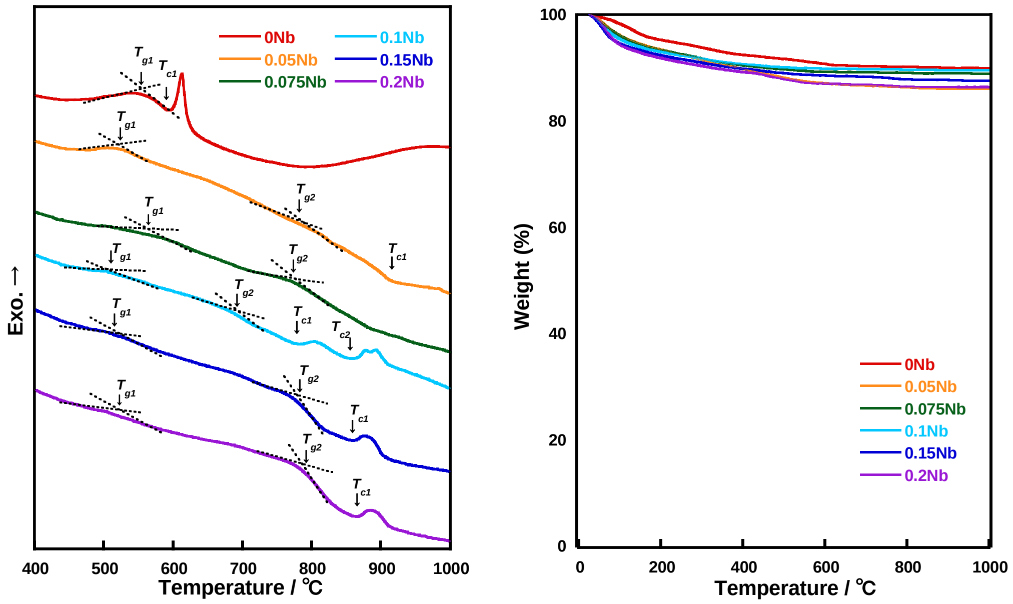

(a) (b)

Figure S2. (a) Differential thermal analysis (DTA) spectra and (b) thermogravimetry of the samples.

Figure S3 (a) shows the DTA spectrum of the samples. The glass transition temperature (Tg) and onset of crystallization temperature (Tc) were obtained from these results. The weight loss of the samples was approximately 11 ~ 14 % at 1000 ºC, as shown in Fig. S3 (b).

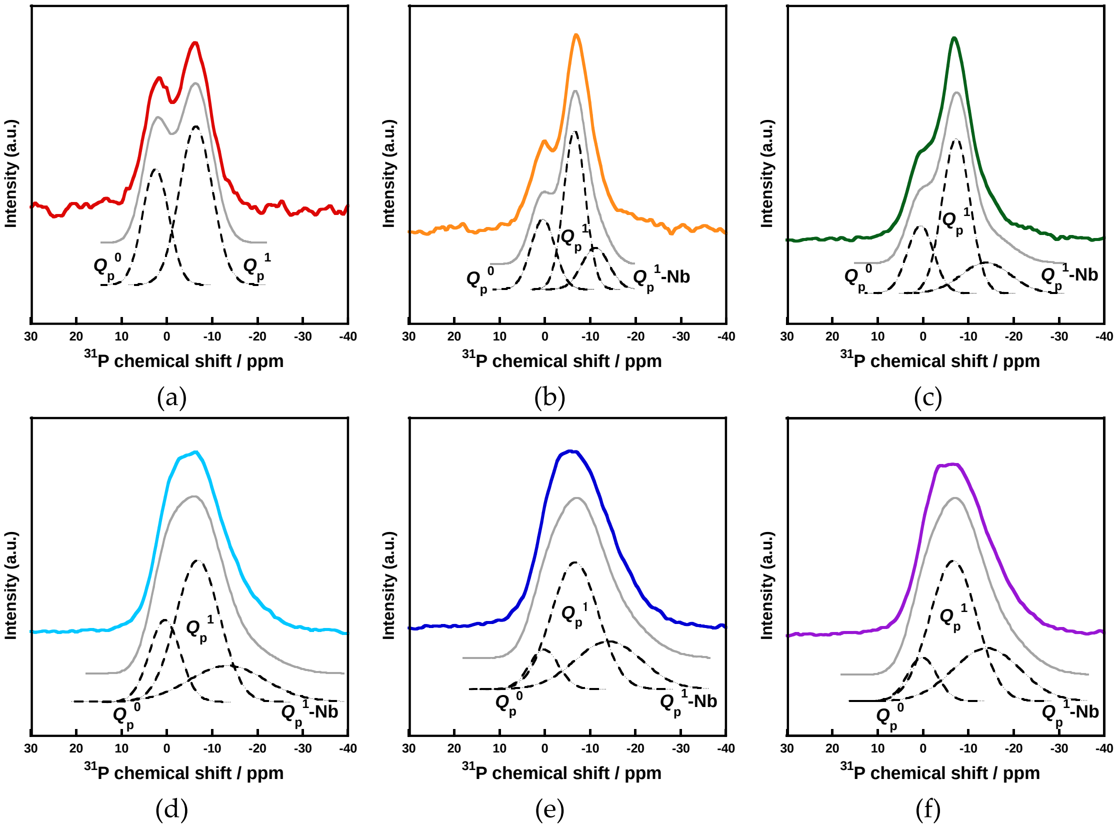

Figure S3. Deconvoluted peaks of ^{31}P MAS-NMR spectra of (a) 0Nb, (b) 0.05Nb, (c) 0.075Nb, (d) 0.1Nb, (e) 0.15Nb and, (f) 0.2Nb. Gray solid lines were fitted waves of *x*Nb, and dotted lines were deconvoluted Q_P^0, Q_P^1 and Q_P^1-Nb peaks.
